# Pleiotropic Ankyrins: Scaffolds for Ion Channels and Transporters

**DOI:** 10.1080/19336950.2022.2120467

**Published:** 2022-09-08

**Authors:** Sharon R. Stevens, Matthew N. Rasband

**Affiliations:** Department of Neuroscience, Baylor College of Medicine, Houston, TX, USA

**Keywords:** Ankyrin, Ankyrin-R, Ankyrin-B, Ankyrin-G, scaffold, ion channels, transporters, pumps, exchange proteins, sodium channels, potassium channels, calcium channels

## Abstract

The ankyrin proteins (Ankyrin-R, Ankyrin-B, and Ankyrin-G) are a family of scaffolding, or membrane adaptor proteins necessary for the regulation and targeting of several types of ion channels and membrane transporters throughout the body. These include voltage-gated sodium, potassium, and calcium channels in the nervous system, heart, lungs, and muscle. At these sites, ankyrins recruit ion channels, and other membrane proteins, to specific subcellular domains, which are then stabilized through ankyrin’s interaction with the submembranous spectrin-based cytoskeleton. Several recent studies have expanded our understanding of both ankyrin expression and their ion channel binding partners. This review provides an updated overview of ankyrin proteins and their known channel and transporter interactions. We further discuss several potential avenues of future research that would expand our understanding of these important organizational proteins.

## Introduction

Ankyrins are membrane-associated scaffolding proteins widely expressed in various tissues and cell types throughout the body including neurons, glial cells, erythrocytes, cardiomyocytes, skeletal muscle cells, and epithelial cells [1,2]. The ankyrin family consists of *ANK1* (*Ank1*, Ankyrin-R, AnkR), *ANK2* (*Ank2*, Ankyrin-B, AnkB), and *ANK3* (*Ank3*, Ankyrin-G, AnkG). Ankyrins connect the submembranous spectrin-based cytoskeleton to the cytoplasmic domain of diverse transmembrane proteins including ion channels, transporters, and cell adhesion molecules. Ankyrins are also critical for membrane organization as they facilitate the recruitment, stabilization, and maintenance of ion channels, transporters, signaling proteins and other membrane proteins to precise subcellular domains. The importance of ankyrins is highlighted by the fact that disruptions to *ANK1-3* cause or are strongly associated with several diseases and disorders, including hereditary spherocytosis, cardiac arrhythmia, Alzheimer’s disease, schizophrenia, autism spectrum disorders, and bipolar disorder [3–7]. A common pathomechanism in these diseases and disorders is that the mutations in ankyrins result in subsequent disruption(s) or mislocalization(s) of ion channels and membrane transporters and/or eliminate the ability of these membrane proteins to be tethered to the spectrin cytoskeleton [8–11]. In this review, we provide an overview of the molecular function and importance of ankyrins and key ion channel and transporter interactions.

Ankyrins are comprised of three main domains, a membrane-binding domain containing 24 ANK repeats, further subdivided into four domains of 6 ANK repeats, D1-D4, which allows them to bind to a wide variety of membrane proteins, a beta-spectrin binding domain that stabilizes ankyrin complexes with the cytoskeleton, and finally a C-terminal regulatory domain [1,3]. This unique structure allows ankyrins to function as important scaffolds by gathering and holding channels and transporters in the right locations and at the right density, thus allowing cells to perform their specific functions.

Ion channels are large membrane proteins found in the lipid bilayer of cells where they function to facilitate the passive movement of ions down electrochemical gradients in response to diverse stimuli like changes in voltage, temperature, stretch, ligands, or pH [12–14]. They regulate fundamental homeostatic cellular processes, such as mediating a cell’s interactions with its environment or regulating a cell’s electrical properties [13,15,16]. Ion channels can be found in both excitable cells like cardiomyocytes and neurons, and in non-excitable cells like epithelial cells. The selective presence of ion channel types within a cell, or within a domain of a cell, enables the cells to perform their unique functions, i.e. muscle contraction in cardiomyocytes, or action potential generation and propagation in neurons. Moreover, different combinations of ion channels can be found in different cell types; and highly specialized cells, like polarized neurons, even contain different combinations and concentrations of channels in several distinct membrane micro-domains [3,17,18].

There are many kinds of ion channels, differentiated by their mechanisms of gating or modulation. These include but are not limited to voltage-gated, ligand-gated, and mechano-sensitive ion channels. A single cell can express several different types of channels, creating vast functional diversity and allowing finely tuned responses to various stimuli. For instance, voltage-gated ion channels respond to perturbations in the membrane potential of a cell and are highly selective for specific ions, including cation channels like voltage-gated sodium (Nav) channels, voltage-gated potassium (Kv) channels, voltage-gated calcium (Cav) channels, or anion channels like voltage-gated chloride channels [13]. Ankyrins are known to have important interactions with these voltage-gated channels [1,8,9,19–25].

Ligand-gated ion channels, also known as ionotropic receptors, use chemical signals to initiate the flux of ions and are generally less selective for specific ions compared to voltage-gated ion channels. AnkG has been proposed to cluster and stabilize α-amino-3-hydroxy-5-methyl-4-isoxazole propionic acid (AMPA) receptors, a type of ligand-gated ion channel [26,27]. Additionally, AnkG has been suggested to interact with and localize cyclic nucleotide-gated (CNG) channels, a nonselective cation signal-gated channel in rod photoreceptors. These channels initiate electrical signals in response to light-induced changes in cGMP concentrations [28]. Mechano-sensitive ion channels respond to changes in mechanical forces by detecting and transducing external mechanical forces into electrical or chemical signals. However, as of yet, no mechano-sensitive ion channel interaction has been reported [29]. Intriguingly though, the potassium leak channels TREK-1 and TRAAK, responsible for the movement of potassium ions out of the cell to set the resting membrane potential in mammalian neurons, are located at nodes of Ranvier where they may interact with ankyrins [30,31].

In addition to ion channels, ankyrins also interact with several membrane transporter proteins, or carrier proteins, that are involved in the movement of ions or other molecules (i.e. bicarbonate (HCO^−^), ammonia (NH_3_), etc.) across biological membranes [32–41]. Transporter proteins can facilitate the diffusion, or passive movement, of these ions or molecules down a concentration gradient, or they can use active transport to move molecules across membranes against concentration gradients. However, transporter proteins require energy to transport molecules if they’re moving molecules against a concentration gradient. Transport proteins can be uniporters that carry one type of molecule against a concentration gradient, symporters that co-transport two molecules unidirectionally, or antiporters that transport two molecules in different directions. In this review, we provide an overview of the molecular function and importance of ankyrins and key ion channel and transporter interactions (these interactions are detailed in Table 1).

**Table 1.** Summary of the ankyrin protein’s known channel and transporter interactions.

Nervous system	Ankyrin	Transporter/Channel interaction(s)	Reference(s)
Inhibitory Neurons: Somatodendritic Domain	AnkR	Kv3.1b, Kv3.3	[10,11]
Neurons: Neuromuscular Junction	AnkR	Nav1.4	[9,95]
Neurons: Nodes of Ranvier *(Secondary Composition)*	AnkR	Nav, Kv3.1b, Kv3.3	[8,10,61]
Neurons: Somatodendritic Domain	AnkB	Cav2.1, Cav2.2	[45]
Neurons: Neuromuscular Junction	AnkB	Nav1.4	[9,95]
Eye: Retina	AnkB	Na^+^/K^+^-ATPase**^†^**, NCX1**^†^**	[40]
Neurons: Axon Initial Segment	AnkG	Nav (Nav1.1, Nav1.2, Nav1.6), KCNQ2/3	[8,19–21,49,96]
Neurons: Nodes of Ranvier *(Primary Composition)*	AnkG	Nav (Nav1.2, Nav1.6; also, Nav1.1, Nav1.8, Nav1.9), KCNQ2/3	[8,17,19,21,51,58,59,97]
Neurons: Somatodendritic Domain	AnkG	AMPAR	[27]
Neurons: Neuromuscular Junction	AnkG	Nav1.4, KCNQ2	[9,95]
Eye: Retina	AnkG	CNG I31	[28]
Brain: Retina, etc.	Ank90	AE3**^†^**	[34,35]
PERIPHERY			
Blood: Erythrocyte	AnkR	Band3 (AE1), Rh, RhAG	[68,69,71,98–100]
Heart: Cardiomyocytes	AnkB	IP3R, Cav1.3, Kir6.2**^†^**,NCX, Na^+^/K^+^-ATPase, SERCA2, RyR**^†^**	[32,33,41,88,90,92,101–103]
Lymphocytes	AnkB	IP3R, RyR**^†^**	[37,89]
Heart: Cardiomyocytes	AnkG	Nav1.5, Kir6.1, Kir6.2	[41,91,104]
Kidney, Lung: Epithelial Cells	AnkG	Na^+^/K^+^-ATPase, ENaC, RhBG**^†^**, kAE1**^†^**	[36,39,79,82,83,100,105]

**^†^**Indicates that the interaction mechanism is not fully understood. For example, the interaction could be indirect, direct interaction has not been explicitly shown, the binding domain is unknown, or there is uncertainty about the ankyrin involved.

## Ankyrins and channels in the nervous system

Ankyrins are critical for ion channel localization and maintenance in the nervous system, and neuronal function. Ion channels allow neurons to rapidly depolarize and repolarize, thus allowing the fast and efficient transmission of neural signals. Important for this is the specific expression and localization of ankyrins in neurons as they give rise to specific profiles of ion channel expression throughout the complex neuronal morphology. This in turn permits these different neuronal regions to have separate functions. For instance, the somatodendritic domain of neurons, expressing AnkB and AnkR, has post-synaptic machinery, such as neurotransmitter receptors and Kv channels, which receive inputs from other cells and modulate excitability. While the axon initial segment, the site of action potential initiation, has high densities of Nav and Kv channels clustered by AnkG to facilitate action potential initiation.

### Neurons: Somatodendritic Domain

**Figure 1.**
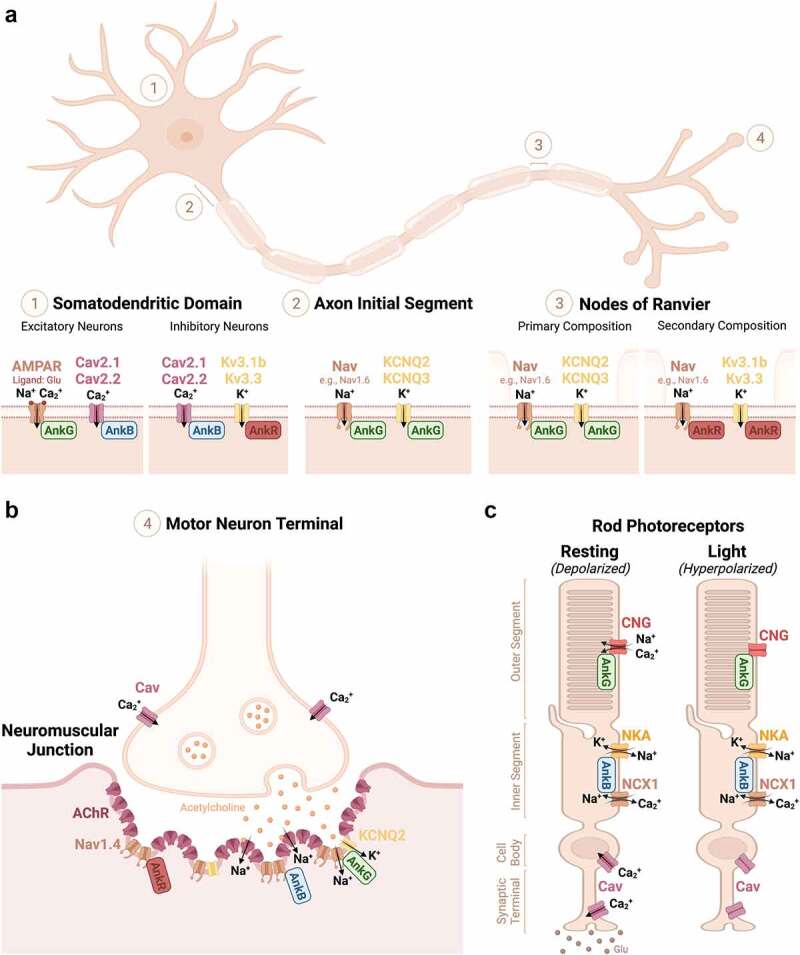
Schematic diagram of ankyrin’s channel and transporter interactions in the nervous system [106]. These include (a) in neurons **(1)** AnkR, AnkB, and AnkG in the somatodendritic domain, **(2)** AnkG at the AIS, and **(3)** AnkG and AnkR at the nodes of Ranvier; (b) AnkR, AnkB, and AnkG at the NMJ; and (c) AnkB and AnkG in rods photoreceptors.

All three ankyrins have been proposed to function in the somatodendritic domain of neurons (Figure 1a, 1). The 190 kDa isoform of AnkG has been reported to function as a perisynaptic scaffold in excitatory dendritic spines where it clusters, stabilizes, and maintains AMPA receptors, including GluA1, though the exact mechanism of binding is poorly understood [27,42]. AMPA receptors, a type of ligand-gated ion channel, are cation permeable ionotropic glutamate receptors that allow the influx of calcium and sodium ions and the efflux of potassium ions to modulate neuronal excitability [43].

More recently, AnkR was shown to be an important ion channel scaffold, critical for proper membrane clustering of Kv3.1b and Kv3.3 in neurons [10,11]. Kv3 channels have fast kinetics and are involved in the rapid repolarization of certain fast-spiking neurons [44]. Notably, Kv3.1 channels are highly expressed in the perisomatic domain of inhibitory parvalbumin-positive neurons, and Kv3.3 channels are highly expressed in the soma and dendrites of cerebellar Purkinje neurons [10,11]. These studies revealed that AnkR links Kv3.1b and Kv3.3 channels to β1 spectrin in forebrain parvalbumin-positive inhibitory neurons in regions like cortex and hippocampus, and links Kv3.3 to β3 spectrin in cerebellar Purkinje neurons. Moreover, loss of AnkR results in a dramatic reduction of these channels in the neuronal membrane causing alterations in the intrinsic excitability of these cells [10,11]. Additionally, these studies reported a novel, nearly identical 6-amino acid motif necessary for AnkR binding, in Kv3.1 (EDCPHI) and Kv3.3 (EDCPAI) potassium channels, not found in the other Kv3 channel subtypes Kv3.2 and Kv3.4 [10,11,44].

AnkB is widely expressed throughout neurons, including in the somatodendritic domain and in axons of unmyelinated neurons. It is also highly enriched at the paranodal junctions of myelinating Schwann cells in the peripheral nervous system. At the somatodendritic domain, AnkB has been proposed to regulate synaptic function by interacting with synaptic molecules including ion channels [45,46]. It has been reported that AnkB is associated with and responsible for the proper targeting of Cav2.1 and Cav2.2 channels, P/Q-type and N-type calcium channels, respectively. These channels are found in neurons and located in several brain regions including the cortex, cerebellum, and spinal cord [45]. Calcium is important for many neuronal cellular processes including synaptic transmission and regulation of synaptic function; these voltage-dependent calcium channels at the presynaptic terminals of neurons allow the flow of calcium ions to control neurotransmitter and/or hormone release [45]. AnkB’s interaction with Cav2.1 and Cav2.2 has been mapped to their DII/DIII cytoplasmic loop [45]. Moreover, a single conserved tyrosine residue in this ankyrin-binding motif has been shown to regulate their interaction: Y797 in Cav2.1 and Y788 in Cav2.2 [45].

### Neurons: Axon Initial Segment and Nodes of Ranvier

The axon initial segment (AIS) is a vital functional site in neurons. Anatomically, the AIS is frequently located in the proximal axon at the axon hillock and is 20–60 µm in length [47]. The AIS has several important functions including initiation of action potentials, modulation of neuronal excitability, and maintenance of neuronal polarity via acting as a boundary between the somatodendritic domain and the axon [47]. Nodes of Ranvier are specialized membrane domains found along myelinated axons throughout the nervous system. Nodes occur as small gaps in the myelin sheath and are responsible for saltatory conduction which allows for fast and efficient action potential propagation [17]. Ankyrins directly interact with Nav channels at the AIS and nodes of Ranvier, these regions have densely clustered Nav channels permitting Na^+^ ion flux, lowering the threshold membrane potential, thus resulting in the initiation and propagation of action potentials down the axon [48]. Ankyrins are critical organizers in these regions and are required for Nav clustering at the AIS and nodes of Ranvier [49,50]. In this section, we provide a brief overview of ankyrins and their channel interactions at the AIS and nodes of Ranvier.

Not surprisingly, AIS and nodes of Ranvier have similar membrane compositions since they perform similar functions: initiate and propagate action potentials (Figure 1a **2, 3**) due to the high densities of Nav and Kv channels [17]. Multiple Nav and Kv channel subtypes can be found at nodes of Ranvier and the AIS, these include Nav1.1, Nav1.2, Nav1.6, KCNQ2, and KCNQ3, with some nodes also expressing Kv3.1, and Kv3.3 [10,17,51–54]. However, in adults the major Nav channel subtype is Nav1.6 and the major Kv channel subtypes are KCNQ2 (Kv7.2) and KCNQ3 (Kv7.3) [17,53,54]. All vertebrate Nav channels, including Nav1.6, have a highly conserved 22-amino acid Na^+^ channel binding motif located near the midpoint of the cytoplasmic loop connecting domains II and III of the Nav α-subunit [17,55,56]. Interaction of Nav channels with AnkG via this motif is necessary and sufficient for targeting these channels to the AIS and nodes of Ranvier [57]. Kv channels also are important at the AIS and nodes of Ranvier as they modulate action potentials. The M-type Kv7 channels Kv7.2, and Kv7.3 (KCNQ2 and KCNQ3) function to stabilize the membrane potential and prevent repetitive excitability. They are clustered in the distal AIS and at nodes of Ranvier by interaction with AnkG [20,58]. The KCNQ2/3 channels occur at these locations as either homomeric or heteromeric complexes that are recruited and stabilized through their interaction with AnkG [20]. Remarkably, KCNQ2/3 channels and AnkG directly interact through a similar binding motif and phosphorylation mechanism as Nav channels; the motif is located in the C3 domain and consists of a 9-amino acid segment that is nearly identical to the Nav channel binding motif [20,59].

Phylogenetic analysis has shown the AnkG binding motif in Nav, KCNQ2 and KCNQ3 channels emerged by convergent evolution after the division of invertebrate and vertebrate lineages with vertebrate orthologs including bony fish, birds, and mammals having this AnkG motif, but worms, insects, or other invertebrates do not [20]. Remarkably, Nav channels and KCNQs have no common genetic ancestor, implying the ankyrin motif in these channels evolved independently from functional need [20]. Consequently, Nav and KCNQ channels must compete for AnkG binding with the outcome of this directly influencing the excitable properties of the AIS and nodes. Post-translational modification of this motif has been shown to impact this, such that phosphorylation of serine residues in the Nav channel ankyrin binding motif increases the affinity of Nav channels for ankyrins by three orders of magnitude, with less efficient AIS targeting of Nav channels seen in phosphorylation incompetent mutant animals [57,60]. Similarly, phosphorylation of KCNQ2/3 by CK2 enhances the binding of their C-terminal anchor domains to AnkG; however, even unphosphorylated Nav channels bind AnkG more strongly than phosphorylated KNCQ2/3 channels [59]. Future studies will be required to further define exactly how the density of Nav and Kv channels is regulated. This is important since the excitability of neurons is defined by the density and distributions of the ion channels in their membranes.

AnkG was once thought to be essential for the formation and maintenance of nodes of Ranvier, however, conditional knockout models where AnkG was specifically removed from nodes have now revealed that AnkG is not essential for Nav channel clustering. Instead, there is a secondary, redundant mechanism to preserve channel clustering and function [8]. This hierarchy of ankyrin-spectrin interactions, where AnkR and β1 spectrin act as a secondary mechanism to cluster nodal proteins if AnkG or β4 spectrin are lost, preserves the function of these critical neuronal sites [8]. At nodes, it was shown that this secondary ankyrin-spectrin complex can bind to the cell adhesion molecule Neurofascin-186, cluster Nav channels, and maintain normal nodal function throughout life [8]. Surprisingly however, and despite their similar functions, AnkR is not able to compensate for a loss of AnkG at the AIS, because it lacks the giant exon required for AIS localization [49,50].

Interestingly, AnkR cannot bind to nodal KCNQ2/3 channels, but no functional difference was seen in mice lacking AnkG at nodes [8]. More recently it was discovered that this may be due to AnkR’s interaction with Kv3-type channels, Kv3.1b, and Kv3.3, which were shown to be recruited to nodes by AnkR and replace KCNQ2/3 channels when AnkG is lost [10]. Previously, Kv3 channel expression was reported at select nodes of Ranvier in the spinal cord, and it was proposed that this occurs through an interaction of Kv3.1 with AnkG [61]. However, the recent results showing direct and strong interaction between AnkR, Kv3.1, and Kv3.3 suggests that AnkR is likely the ankyrin responsible for the select nodal localization [10]. This is intriguing since AnkR has also been reported to be present at some nodes [8]. Conversely, it has been reported that AnkG governs the axon-dendrite targeting of Kv3.1 *in vitro* through the interaction of Kv3.1´s N-terminal T1 domain and a C-terminal axonal targeting motif in AnkG [62]. Thus, in the future, it will be interesting to further elucidate the interplay of ankyrins and Kv channels at nodes, as well as determine if Kv3 channels form homomeric and/or heteromeric complexes like KCNQ2/3.

Though AnkG is a critical scaffold for several ion channels and other molecules necessary for the function of the AIS, it is not the only mechanism as other channels found at the AIS have other clustering mechanisms. Notably, there are several ankyrin-independent mechanisms responsible for the clustering of channels and receptors at sites of inhibitory synapses along the AIS, including the postsynaptic density-93 (PSD-93) protein, a domain-containing membrane-associated guanylate kinase (MAGUK), which clusters Kv1 channels (Kv1.1, Kv1.2, Kv1.4, and Kv2) and Gephyrin which is involved in the clustering of γ-aminobutyric acid type A receptors (GABA_A_Rs) [63–65]. Additional details on the molecular composition of the AIS and nodes of Ranvier can be found in Leterrier (2018) [18] and Rasband and Peles (2021) [17].

#### Neuromuscular Junction

The neuromuscular junction (NMJ) is a highly specialized synapse and site of neurotransmission that occurs between the nerve terminals of motor neurons and muscle fibers; synaptic transmission from the motor neuron to the muscle results in muscle contraction. This junction contains three main types of channels, (1) Cav channels at the presynaptic terminal of the motor neuron, (2) acetylcholine receptors (AChRs), a type of ligand-gated cation channel clustered at the crests of the synaptic folds, and (3) Nav channels clustered in the troughs of the synaptic folds (Figure 1b).

While the mechanisms responsible for the clustering and function of AChR at the NMJ have been well characterized (see Li et al., 2018 for a comprehensive review) [66], the mechanisms regulating Nav channel clustering and the functional significance of its localization at the NMJ were only recently reported. Surprisingly, all three ankyrins are found at the NMJ, and all three are capable of clustering muscle-specific Nav1.4 channels [9,66]. Loss of any single ankyrin does not impair Nav channel clustering. Similarly, loss of two ankyrins results in only a partial loss of Nav channels, and loss of any single or pair of ankyrins does not result in any motor or synaptic deficits [9]. However, when all three ankyrins are removed by genetic deletion, there is a total loss of NMJ Nav channel clustering and impaired ability to resist synaptic fatigue [9]. Together, this reveals the functional importance of Nav channels at the NMJ and highlights another example of the remarkable biological redundancies of ankyrins.

Interestingly, this was not the case for KCNQ2 channels. These Kv channels are clustered at a subset of NMJs. Like at the node of Ranvier, loss of AnkG alone is sufficient to block KCNQ2 localization while loss of AnkB and/or AnkR has no effect [9]. One intriguing possibility in the latter situation is that other Kv channels may be recruited to the NMJ when AnkG is absent, similar to what occurs at nodes of Ranvier. Future experiments will need to explore this possibility to determine if other types of Kv channels present at the NMJ, if a switch in Kv channel type (e.g. from KCNQ2 to Kv3 channels) occurs when AnkG is lost, and to determine the functional importance of Kv channels at the NMJ.

In addition to being expressed in the troughs of the synaptic folds of the NMJ, ankyrins have also been reported in skeletal muscle fibers, though little is known about their function in these fibers. Specifically, AnkG and AnkR were reported in the Z-lines between adjacent sarcomeres [24]. A 100 kDa isoform of AnkG was described as having a reticular cytoplasmic distribution and colocalized at sarcoplasmic reticular structures with the Ca^2+^-ATPase transporter, SERCA1, while AnkR was reported to have a punctate sarcolemma localization [24], indicating that ankyrins may also be involved in organizing the membrane of myofibers, but little else is known making this another potentially interesting avenue for further research. Nevertheless, these interactions outside of the NMJ may not be critical since, as described above, mice lacking all three ankyrins only show neuromuscular synapse fatigue and no degeneration [9].

#### Retina: Rod Photoreceptors

In the retina, AnkB and AnkG are expressed in rod photoreceptors, one of the two types of photoreceptors used to convert photons from visual stimuli to chemical and electrical signals necessary for neural processing. Rods, in comparison to cones, are more sensitive to light and are concentrated in the outer edges of the retina. Together, these characteristics allow rods to be the primary photoreceptor in low light and peripheral vision. Rods are highly polarized cells with three main compartments: a synaptic terminal, an inner segment, and an outer segment; each of these compartments has a specialized membrane organization of ion channels and transporters, including Cav channels, cyclic nucleotide-gated (CNG) channels, Na^+^/K^+^-ATPase, and Na^+^/ Ca^2+^ exchanger (NCX1) [28,40,67].

The transduction of visual stimuli in rods is based on their inhibition, or hyperpolarization, as the resting state in these cells is depolarized and allows spontaneous neurotransmitter release [67]. Rods maintain this resting state by the influx of cations via cyclic nucleotide-gated (CNG) channels in the outer segments, the inner segment partially balances this with the Na^+^/K^+^-ATPase, a transporter that effluxes Na^+^and influxes K^+^. The inner segment also contains the NCX1, which influxes Na^+^and effluxes Ca^2+^ ions. The overall depolarization of the rod caused by Na^+^ influx from the CNG channel and NCX1 results in activation of Cav channels at the cell body and synaptic terminal. This influx of Ca^2+^ stimulates the tonic release of neurotransmitters (e.g. glutamate). Upon light activation of the rod, CNG channels are inactivated, NCX1 continues to export Ca^2+^, the cell becomes hyperpolarized, and Cav channels close preventing the release of inhibitory glutamate [40,67].

The different distributions of ankyrin proteins, with AnkB localized to rod inner segments and AnkG localized to rod outer segments, is an important molecular mechanism driving the organization of the transporters and channels in rods [28,40] (Figure 1c). AnkG’s interaction with the β1-subunit in the C-terminal domain of CNG is required for CNG to be localization to rod outer segments [28]. Meanwhile, AnkB is required to restrict the localization of NCX1 and Na^+^/K^+^-ATPase, with the loss of AnkB resulting in a significant reduction of these transporters in rod inner segments [40]. Cones, photoreceptors that respond well in bright light and are responsible for color vision, have a similar transduction mechanism utilizing CNG channels, Na^+^/K^+^-ATPases, and Na^+^/ Ca^2+^ exchangers; thus, it will be important to determine if ankyrins are important scaffolds in these cells as well.

Furthermore, an ankyrin protein fragment ANK90 has been shown to interact with the Cl^−^/HCO_3_^−^ anion exchanger AE3. ANK90 is expressed in the brain including in neurons and Müller glia in the retina, and the heart [34,35]. While it remains to be fully elucidated which ankyrin is responsible, AnkR interacts with AE1 in erythrocytes and would be an interesting candidate to explore further.

## Ankyrins and channels in the periphery

### Erythrocytes

**Figure 2.**
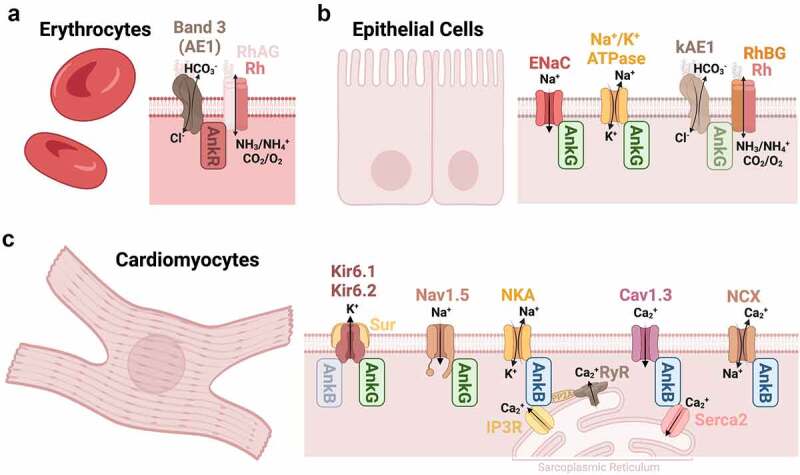
Schematic diagram of ankyrin’s channel and transporter interactions in the periphery [106]. These include, (a) AnkR in erythrocytes, (b) AnkG in epithelial cells, and (c) AnkB and AnkG in cardiomyocytes.

AnkR (also known as erythrocyte ankyrin) was discovered due to its critical role in red blood cells where it maintains the cell’s structural integrity through its link between β1 spectrin and the cytoplasmic domain of Band 3, also known as AE1, and the Rh complex [68,69]. The membrane of erythrocytes is organized into a polygonal network formed by α1/β1 spectrin tetramers linked to actin filaments [70]. This cytoskeletal structure is coupled to the membrane by β1 spectrin interacting with AnkR, which is then bound to the membrane by interaction with Band 3 and Rh proteins [69–72] (Figure 2a).

The flexibility and structure provided to the membrane by the cytoskeleton provide the support necessary for erythrocytes to survive the mechanical stresses of circulation [70]. Moreover, these interactions between AnkR, membrane transporters, and the cytoskeleton are critical for erythrocyte health. In fact, hereditary spherocytosis (HS), a severe form of hemolytic anemia, results from defects in the erythrocyte proteins AnkR, β1 spectrin, α1 spectrin, Band 3, and protein 4.2, with dominant and recessive mutations in AnkR accounting for the majority of cases [73].

Band 3 is an anion exchange protein that mediates the active exchange of Cl^−^/HCO_3_^−^ in erythrocytes [74]. Several critical regions have been identified for the interaction of AnkR and Band 3, these include the third (D3) and fourth (D4) repeat domain of AnkR, and residues 63–73 on Loop 1 and 175–185 on Loop 2 of Band 3 [75,76]. Whereas the second repeat domain (D2) of AnkR has been found to directly interact with the C-terminal, cytoplasmic, domain of the Rh and RhAG protein complex [69]. The Rh proteins are transporter proteins in erythrocyte membranes that mediate ammonium transport [69]. Interestingly, there is also evidence supporting the Rh complex may function as a gas channel, facilitating the movement of CH_3_/NH_2_/NH_3_ and CO_2_/O_2_ [69,77].

### Epithelial Cells

Epithelial cells are dynamic barriers that separate physiological compartments and are found in several organs, including the kidneys and lungs. They are armed with a wide variety of membrane pumps, transporters, and ion channels, which allow fluid balance, filtration, absorption, and trafficking of metabolites and electrolytes critical for proper function. Using TEM it was determined that AnkG is closely associated with both the apical and basolateral membranes of the kidney, however, its precise localization in the lungs remains to be determined [78]. However, it is known that AnkG is a critical scaffold for several types of transporters in epithelial cells. For example and like AnkR in erythrocytes, AnkG has been linked to the ammonium transporter Rh proteins, in particular, RhBG in lung and kidney epithelial cells [79]. Furthermore, the kidney anion exchange protein 1 (kAE1), similar to Band 3/AE1 in erythrocytes, is part of the epithelial Rh molecular complex [36]. Inhibition of AnkG slows down membrane targeting and decreases the stability of RhBG [79]. Moreover, studies have shown that the transport activity of both kAE1 and RhBG in kidney epithelial cells depends on association with AnkG, for Cl^−^/CHO_3_^−^ and NH_3_, respectively [36,79,80]. Interestingly, phosphorylation of the C-terminal tail of RhBG at Tyrosine 429, adjacent to the AnkG interaction domain (^419^FDL^421^), regulates NH_3_ transport activity, such that dephosphorylation allows RhBG to be anchored to the membrane via AnkG and activation of channel function [79]. A similar mechanism has been proposed for kAE1, though direct interaction of AnkG with kAE1 has yet to be shown [79]. However, kAE1 does bind the renal epithelial cell transporter Na^+^/K^+^-ATPase, another known AnkG binding partner [81,82]. Na^+^/K^+^-ATPase has been reported to bind to two independent domains on AnkG (190 kDa), one of which is located in the distal 12 ANK repeats, and the other in the spectrin binding domain [82].

Finally, AnkG also regulates the membrane insertion and activity of epithelial sodium channels (ENaC) in the kidney and lungs that transport Na^+^ in response to hormonal cues to maintain blood pressure homeostasis [39,83]. The interaction of AnkG with ENaC and RhBG depends on phosphorylation, similar to AnkG and Nav and KCNQ channels in the nervous system [79,84]. Remarkably, AnkG is also required for the development and assembly of lateral membrane domains in epithelial cells, as depletion of 190 kDa AnkG in human bronchial epithelial cells results in significant loss of lateral membrane biogenesis [85]. Thus, in epithelial cells, AnkG functions as a critical scaffold for transporters and channels which allow the flux of Cl^−^, CHO_3_^−^, NH_3_, K^+^, and Na^+^, and as an important protein for the formation of epithelial cell lateral membranes (Figure 2b).

### Cardiomyocytes

Proper rhythmic contraction of the heart requires finely-tuned control of ion fluxes and calcium homeostasis. Ankyrins are important for the correct localization of calcium transporters, such that AnkB and AnkG organize distinct molecular complexes in cardiomyocytes (Figure 2c). AnkB binds to and localizes Na^+^/K^+^-ATPase, NCX1, a Na^+^/ Ca^2+^ exchanger, and Cav1.3 channels at the cardiomyocyte membrane [32,33,86]. AnkB is also known to interact with inositol triphosphate receptors (IP3Rs) [38], is reported to bind to SERCA2 [87], a Ca^2+^-ATPase transporter, and is thought to indirectly help regulate ryanodine receptors (RyRs), three important Ca^2+^ transporters found in the sarcoplasmic reticular membrane of cardiomyocytes [88]. Interestingly, AnkB has also been reported to directly bind IP3R and indirectly bind RyR in the sarcoplasmic reticulum of lymphocytes [37,89,90].

On the other hand, AnkG has been shown to interact with the inwardly-rectifying potassium channels Kir6.1 and Kir6.2 and Nav1.5 in cardiomyocytes [3,41,91]. Kir channels are a subtype of Kv channels but are unique in that they associate with sulphonylurea receptors (Surs) to form ATP-sensitive K^+^ channels which allow a large influx with minimal efflux of K^+^ [41,92]. While AnkB has been shown to have the ability to interact with the Kir6.2, a type of inwardly-rectifying potassium channel, *in vitro*, it is debated if this interaction occurs in cardiomyocytes as more recent data using super-resolution microscopy has shown they are not co-localized [41]. Nav1.5 channels are the principle Nav channels in the heart and directly interact with AnkG [25,93]. Furthermore, DNA sequencing of patients with Brugada Syndrome, a type of arrhythmia caused by mutations in *SCN5A* (which encodes Nav1.5), found that substitution of a highly conserved glutamic acid with lysine (E1053K) in the ankyrin binding motif in the DII-III loop of Nav1.5 channels causes loss of AnkG binding and the subsequent failure of localization of Nav1.5 channels to the membrane of cardiomyocytes [25,93]. Since AnkG is a critical scaffold for the targeting and organization of Nav channels in plasma membranes of cardiomyocytes, it will be interesting to determine if other ankyrins can compensate for disruptions to AnkG as has been shown in the nervous system with AnkR at nodes of Ranvier and AnkR and AnkB at the NMJ [8,9].

## Conclusions

Our understanding of the molecular mechanisms for how ankyrins regulate and interact with ion channels, receptors, and transporters has expanded greatly in recent years. For example, just in the previous two years, it was discovered that AnkR cannot compensate for the loss of AnkG at the AIS to cluster Nav channels, even though it does so at nodes of Ranvier, because it lacks the giant exon needed for axonal targeting [94]. We now know that at nodes of Ranvier, AnkR recruits Kv3.1b and Kv3.3 to replace KCNQ2/3 channels when AnkG is lost and the specific motif in Kv3.1b that interacts with AnkR has been identifed [10]. Furthermore, Nav channels were found to prevent synaptic fatigue at the NMJ and were found to be clustered by all three ankyrins [9]. However, KCNQ2 channels, also found at the NMJ, require AnkG to be localized [9]. Lastly, it was recently discovered that AnkR is critical for proper clustering of Kv3.1b and Kv3.3 in the perisomatic membrane in a subset of forebrain interneurons, and loss of AnkR impairs the intrinsic excitability of these neurons [10,11]. This review highlights these new findings and illustrates examples where ankyrins play essential roles in the clustering, localization, and function of ion channels and transporters. Importantly, although some ankyrin binding motifs are highly conserved (e.g. the ankyrin-binding motif in Na^+^ channels) the studies described here illustrate the diversity of motifs and interactions that can occur between ion channels, receptors, and ankyrins. Thus, a general principle highlighted in this review is that ankyrins serve a common purpose in diverse cellular contexts to cluster and maintain different ion channels, transporters, and receptors in subcellular domains. Future studies will be needed to further define the structural bases of these interactions, how they may be modulated by post-translational modifications or signaling pathways, and how the expression levels of ankyrins and the ion channels they bind are coordinately regulated. We predict that future research focused on ion channels, transporters, and their regulation and localization by ankyrins will yield important insights into the mechanisms regulating cellular excitability in both health and disease.
